# Associations of nonconforming gender expression and gender identity with bullying victimization: an analysis of the 2017 youth risk behavior survey

**DOI:** 10.1186/s12889-022-13071-6

**Published:** 2022-04-05

**Authors:** Qiguo Lian, Ruili Li, Zhihao Liu, Xiaona Li, Qiru Su, Dongpeng Zheng

**Affiliations:** 1 NHC Key Lab. of Reproduction Regulation (Shanghai Institute for Biomedical and Pharmaceutical Technologies), Fudan University, Shanghai, China; 2 Capital Institute of Pediatrics, Beijing, China; 3 Jiangsu Provincial Center for Disease Control and Prevention, Jiangsu, China; 4 Beichen District Center for Disease Control and Prevention, Tianjin, China; 5 Children’s Healthcare & Mental Health Center, Shenzhen Children’s Hospital, 7019 Yitian Road, Shenzhen City, Guangdong, 518038 China; 6 Kangjian Community Health Service Center, 88 Jiang’an Road, Shanghai, China

**Keywords:** Gender nonconformity, Transgender identity, Bullying victimization

## Abstract

**Purpose:**

Although gender nonconformity (GNC) and transgender identity are both linked to bullying victimization, few studies have examined them with bullying victimization simultaneously. Using a sample of Youth Risk Behavior Survey, we investigated the associations of GNC and transgender identity with bullying victimization within the same study.

**Methods:**

We analyzed data from the cross-sectional school-based Youth Risk Behavior Survey in 2017 (*n* = 25,378). The exposures were GNC and transgender identity. The main outcomes were traditional victimization, cyber victimization, and combined victimization. We calculated adjusted prevalence ratios (APRs) with 95% confidence intervals (CIs) using Poisson regression models.

**Results:**

There were 22.15% of high school students with GNC, and 1.61% identified themselves as transgender. GNC is associated with traditional (APR,1.45;95%CI, 1.21–1.73), cyber (APR,2.00; 95%CI, 1.66–2.40) and combined victimization (APR,1.61;95%CI, 1.42–1.83) respectively among assigned male at birth (AMAB) students only. Transgender male and female students are both at higher risk of bullying victimization for all the three outcomes than cisgender peers.

**Conclusions:**

AMAB GNC and transgender identity are associated with a higher risk of bullying victimization. Providing support systems and celebrating gender diversity within and outside schools are important.

## Background

Bullying victimization negatively affects the health of about 20% of American high school students [1], including suicidal thoughts and behaviors [2], making it a serious public health concern for adolescents in the United States and a high priority in Healthy People 2020 [3]. Adolescents with gender nonconformity (GNC) report elevated rates of negative health outcomes, including bullying victimization [4–7].

Preventing school bullying is a fundamental human rights issue, and any form of bullying infringes the fundamental right to education. Sexual and gender minority adolescents are more likely to experience school bullying [8]. Although many studies consider lesbian, gay, bisexual (LGB), and transgender(T) as an umbrella term LGBT, “LGB” and “T” are different in nature: the former refers to sexual orientation while the latter refers to gender identity. A growing body of research suggests that transgender adolescents experience substantial disparities in bullying victimization [7, 9–12]. For example, in 2012, a New Zealand nationally representative survey found that transgender students and those unsure about their gender identity were more prone to be bullied than their cisgender peers [12].

There are also a few studies that have examined bullying victimization across the full spectrum of gender expression [4, 13–15]. For example, a study based on the 2013 Youth Risk Behavior Survey (YRBS) suggested that there was a linear association between GNC score and bullying victimization among U.S. high school students, and each additional unit of GNC score is associated with a 15% risk increase in traditional or cyber victimization [15].

Transgender identity is gender identity that differs from assigned sex at birth (ASAB), while GNC is gender expression (eg, in clothing or mannerisms) that differs from societal expectations of the gender (eg, boys like blue and girls like pink) [6]. Considering that transgender students often have experiences that highly overlap with those of cisgender, gender nonconforming peers [6], it is important to investigate whether the associations of bullying victimization with gender identity are similar to the findings with gender nonconforming students. Few existing studies, however, were available on the measures of both gender expression and gender identity. Fortunately, both GNC and transgender identity were collected in a partial sample of 2017 YRBS, which provides us a unique opportunity to address this gap.

Leveraging the data from 2017 YRBS, the objective of this study was to investigate the association of bullying victimization with GNC and transgender identity among U.S. high school students.

## Methods

This cross-sectional secondary data analysis of a sample of YRBS was not considered human subject research, because YRBS datasets are de-identified and publicly available (https://www.cdc.gov/healthyyouth/data/yrbs).

### Data sources

The YRBS, developed by the U.S. Centers for Disease Control and Prevention, is conducted every two years to collects priority risk behaviors, including bullying victimization, that contribute to social problems, disabilities, and even death among high school students in United States [1].

About 20 large urban school districts in the U.S. conduct YRBS biennially using a 2-stage cluster sampling strategy to guarantee a representative sample of students in grades 9–12 within each jurisdiction [1, 6]. In 2017, nine U.S. school districts included the items to measure gender expression and gender identity simultaneously, and the data from these 9 school districts (1 in Illinois, 1 in California, 1 in Florida, and 6 in New York) were combined into a single sub-data (*N* = 25,378) for further analysis in the present study. The overall response rates ranged between 61 and 89%[1], and the sample size ranged between 938 and 10,191 students. The surveys were reviewed and approved within each jurisdiction using its local procedure. Detailed information concerning the large urban school district YRBS has been provided elsewhere [16].

### Measures

The perceived gender expression was measured with one validated item developed for adolescents in school contexts [17]. Students were asked: “A person’s appearance, style, dress, or the way they walk or talk may affect how people describe them. How do you think people at school would describe you?” Response options followed with a 7-point scale: “very feminine,” “mostly feminine,” “somewhat feminine,” “equally feminine and masculine,” “somewhat masculine,” “mostly masculine,” and “very masculine.” A continuous GNC score that measures the degree of nonconformity to gender norms was created by combining the responses to the gender expression and ASAB, with 1 indicating most gender conforming (very feminine girls and very masculine boys) and 7 indicating most gender non-conforming (very masculine girls and very feminine boys) [6]. Then a binary GNC variable was generated: gender conformity (GC) (GNC score: 1–3) and GNC (GNC score: 4–7) (Table 1) [6].

**Table 1:** Definitions of variables, questionnaire items and analytic coding

Terminology	Definition ^a^	YRBS questionnaire item	Analytic coding
**Sex and gender**			
Assigned sex at birth	An individual’s biological status as male, female, or something else, which is associated with physical attributes, such as anatomy and chromosomes	What is your sex?	Assigned female at birth vs assigned male at birth
Sexual orientation	One’s enduring sexual attraction to male partners, female partners, or both	Which of the following best describes you?	Heterosexual, gay or lesbian, bisexual, not sure
Gender expression	The manner in which an individual chooses to present their gender to others through physical appearance and behaviors, e.g., style of hair or dress, voice, or movement	A person’s appearance, style, dress, or the way they walk or talk may affect how people describe them. How do you think other people at school would describe you?	Gender conformity vs gender nonconformity
Gender identity	An individual’s sense of their self as male, female, transgender, or something else	Some people describe themselves as transgender when their sex at birth does not match the way they think or feel about their gender. Are you transgender?	Cisgender, transgender, not sure
**School bullying**			
Traditional bullying	Physical bullying (e.g., hitting, pushing, and kicking), verbal bullying (e.g., name-calling and teasing in a hurtful way), and relational bullying (e.g., social exclusion and spreading rumors)	During the past 12 months, have you ever been bullied on school property?	Yes vs no
Cyber bullying	An extension of traditional bullying that performed via electronic means such as mobile/cell phones or the internet	During the past 12 months, have you ever been electronically bullied? (Count being bullied through texting, Instagram, Facebook, or other social media.)	Yes vs no
Combined bullying	Traditional or cyber forms of bullying	Computed variable: being bullied traditionally or electronically during the past 12 months	Yes vs no

^a^Definitions related sex and gender adapted from American Psychological Association (https://www.apa.org/pi/lgbt/resources/sexuality-definitions.pdf; accessed June 2019). Definitions related bullying adapted from “School bullying: development and some important challenges” by Dan Olweus, [18], Annual Review of Clinical Psychology, 9(1):751–780.

Gender identity was measured with the item: “Some people describe themselves as transgender when their sex at birth does not match the way they think or feel about their gender. Are you transgender?” Response options were “No (Cisgender)”, “Yes, I am transgender (Transgender)”, and “Not sure if I am transgender (Not sure)”.

Traditional victimization was measured with the item: “During the past 12 months, have you ever been bullied on school property?” (No = 0, Yes = 1). Cyber victimization was measured with the item: “During the past 12 months, have you ever been electronically bullied? (Count being bullied through texting, Instagram, Facebook, or other social media.)” (No = 0, Yes = 1). Then combined victimization was computed to indicate if the participants experienced traditional or cyber bullying during the past 12 months before the survey (No = 0, Yes = 1).

Demographic characteristics that were assessed in this study included ASAB (assigned female at birth (AFAB) and assigned male at birth (AMAB)), race/ethnicity (White, Black or African American, Hispanic/Latino and all other races), sexual orientation (heterosexual, gay or lesbian, bisexual, and unsure), and grade (9th, 10th, 11th, and 12th).

### Statistical analysis

We estimated the prevalence of GNC and transgender identity and tested the differences by ASAB using χ^2^ statistics. We investigated associations of GNC and non-cisgender identity with bullying victimization, controlling for the grade, race/ethnicity, and sexual orientation. We calculated the adjusted prevalence ratios (APRs) with 95% confidence intervals (CIs) using Poisson regression models [19], with GC and cisgender identity as reference groups respectively, because APRs are more interpretable than adjusted odds ratios in cross-sectional studies [19, 20].

All statistical analyses were weighted and performed using Stata/SE 15.1 (StataCorp LLC) to account for the complex survey design. The tests were considered to be statistically significant if two-tailed *P* < 0.05.

## Results

The weighted percentage (unweighted number) of participants in the current study was 49.17% (*n* = 13,035) AFAB and 50.83% (*n* = 12,343) AMAB. There were 23.14% (*n* = 5,728) of students identified as sexual minority (lesbian or gay: 3.13%, bisexual: 7.61%, and unsure: 1.24%). For gender identity, 3.31% (*n* = 728) identified as transgender or unsure (transgender: 1.61% and unsure: 1.70%). And 22.15% (*n* = 5,224) of students were labeled with GNC. As shown in Table 2, sexual minority was more prevalent among AFAB than AMAB participants (AFAB: 28.63% vs. AMAB: 17.80%, *P* < 0.001) while transgender or unsure identity was more prevalent among AMAB than AFAB participants (AFAB: 2.88% vs. AMAB: 3.73%, *P* < 0.05). No statistically significant ASAB differences were observed for GNC.

**Table 2:** Descriptive statistics of demographic characteristic

Demographic Characteristic	ASAB	
	AFAB, n (%)^a^	AMAB, n (%)^a^
**Total**	13,035(100)	12,343(100)
**Race/ethnicity**		
White	1,746(17.05)	1,469(16.49)
Black or African American	2,751(27.94)	2,755(30.01)
Hispanic/Latino	5,801(38.45)	5,325(36.34)
All other races	2,247(16.57)	2,153(17.15)
**Grade**		
9th	3,408(27.58)	3,234(28.62)
10th	3,720(26.48)	3,371(26.72)
11th	2,942(23.75)	2,671(22.99)
12th	2,863(22.19)	2,911(21.67)
**Sexual orientation**		
Heterosexual	9,031(71.37)	9,805(82.20) ^***^
Lesbian/gay	405(3.28)	346(2.98)
Bisexual	1,565(11.73)	417(3.61)
Not sure	1,650(13.62)	1,345(11.21)
**Gender expression**		
Gender conformity	9,716(78.44)	8,648(76.13)
Gender nonconformity	2,606(21.56)	2,618(23.87)
**Gender identity**		
Cisgender	11,214(97.12)	10,260(96.27) ^*^
Transgender	137(1.14)	197(2.08)
Not sure	196(1.75)	198(1.65)

*ASAB* assigned sex at birth, *AFAB* assigned female at birth, *AMAB* assigned male at birth

^a^Sample number is unweighted, percentage is weighted. ^*^P < 0.05, ^***^ *P* < 0.001

ASAB differences were observed in the associations of GNC with three outcomes of bullying victimization. Table 3 shows that associations of GNC with traditional victimization (APR,1.45;95%CI, 1.21–1.73), cyber victimization (APR,2.00; 95%CI, 1.66–2.40) and combined victimization (APR,1.61;95%CI, 1.42–1.83) were all statistically significant among AMAB students only. None of the three outcomes of bullying victimization was associated with GNC among AFAB students.

**Table 3:** Associations between gender expression and bullying victimization

Bullying Victimization	Gender expression		
	GC, %^a^	GNC, %^a^	APR (95%CI)
**AFAB**	**AFAB GC**	**AFAB GNC**	
Traditional victimization	16.07	18.41	1.09(0.92–1.29)
Cyber victimization	14.46	18.55	1.17(0.98–1.40)
Combined victimization	22.13	26.24	1.08(0.94–1.24)
**AMAB**	**AMAB GC**	**AMAB GNC**	
Traditional victimization	12.54	19.96	1.47(1.23–1.75) ^***^
Cyber victimization	8.67	20.89	2.00(1.65–2.42) ^***^
Combined victimization	16.24	30.33	1.62(1.43–1.84) ^***^

*AFAB* assigned female at birth, *AMAB* assigned male at birth, *GC* gender conformity, *GNC* gender nonconformity, *APR, APR* adjusted prevalence ratio (adjusted for race, grade, and sexual orientation, with gender conformity as the referent group)

^a^Percentage is weighted. ^***^ *P* < 0.001

Table 4 shows that students who were transgender male, transgender female, and unsure about their gender identity all reported higher risks of three outcomes of bullying victimization compared with their cisgender peers. And all the associations were statistically significant. For example, 13.47% of cisgender male students reported being bullied traditionally, while the prevalence rose up to 33.38% among transgender males. Transgender males were more likely to experience traditional victimization than their cisgender male peers (APR, 2.10;95%CI, 1.42–3.12).

**Table 4:** Associations between gender identity and bullying victimization

	Bullying victimization		
	Traditional	Cyber	Combined
Cisgender male, %^a^[reference]	13.47	10.06	17.66
Transgender male, %^a^	33.38	33.52	42.22
APR (95%CI)	2.10(1.42–3.12)^***^	2.32(1.51–3.56)^***^	1.82(1.28–2.58)^**^
Cisgender female, %^a^[reference]	15.92	14.99	22.27
Transgender female, %^a^	20.08	27.77	36.00
APR (95%CI)	1.53(1.08–2.16)^*^	1.63(1.17–2.27)^**^	1.63(1.22–2.16)^**^
Cisgender, %^a^ [reference]	14.78	12.62	20.07
Not sure, %^a^	31.31	28.22	46.55
APR (95%CI)	1.74(1.31–2.31)^***^	1.67(1.29–2.16)^***^	1.87(1.58–2.22)^***^

*APR* adjusted prevalence ratio (adjusted for race, grade, and sexual orientation)

^a^Percentage is weighted. ^*^P < 0.05, ^**^P < 0.01, ^***^ *P* < 0.001

## Discussion

Reducing bullying of transgender students is a high-priority public health issue, and may be considered to become a core Healthy People 2030 objective when reliable baseline data are available [21], and essential in order to achieve the Sustainable Development Goals for children and adolescents. This study expands the existing literature on the differences and similarities of the associations of bullying victimization with GNC and transgender identity. We found that the impact of gender nonconformity is different depending on ASAB (but not on transgender identity) after adjusting the grade, race/ethnicity, and sexual orientation.

A previous study revealed that a higher score on verbal bullying scale demonstrated a linear increase with increasing GNC scores for both ASAB, while this linear association was stronger for AMAB students than for AFAB students [22]. In our study, GNC is regarded as more acceptable for AFAB students than for AMAB students, while transgender identity is discriminated against for both male and female students.

These findings indicate the ASAB differences in discrimination, stigmatization, and marginalization related to GNC. The major reason for the ASAB differences, as previous studies indicated [23–25], is the social negative views on GNC are meted out unequally to the AMAB and AFAB students. AFAB GNC is often treated much less harshly than AMAB GNC although both AMAB GNC and AFAB GNC are less valued among their peers [23, 26] and predict low self-perceived social competence [27]. This phenomenon roots in higher tolerance of gender non-conforming behaviors among AMAB students [28] and highly valued masculinity in our society [29]. Thus the prejudice toward students with GNC largely depends on their ASAB and gender expression [23]. Besides, the ASAB bias in reporting bullying could possibly attenuate the ASAB difference. Compared with gender nonconforming AFAB peers, AMAB students with GNC may be less likely to report being bullied, given that masculinity is associated with a reticence to disclose being bullied for fear of being seen as ‘weak’ [6].

Important similarities and differences emerged across gender when we examined the association between transgender identity and bullying victimization. We found that compared with cisgender peers, transgender students were more likely to be bullied, traditionally and electronically, among both male and female students. Additionally, students who were unsure about their gender identity were also at higher risk of experiencing traditional or cyber bullying.

Prior research has found that compared with cisgender peers, transgender students experienced higher risk of bullying victimization [7] and gender-based bullying victimization [9]. The unique strength of the present study is its capability to compare the associations of bullying victimization with GNC and transgender identity, given that transgender students often shared similar risk factors and experiences with peers with GNC [6]. We noted that AMAB GNC is associated with a higher risk of bullying victimization, while AFAB GNC is not. However, transgender identity is associated with bullying victimization for not only males but also females. This finding highlights that AMAB GNC and transgender identity could be monitored as risk factors of bullying victimization among U.S. high school students.

### Implications

Our findings appear to have essential practical implications. Transgender youth in U.S. are facing an unprecedented number of anti-transgender legislations. In just the first week of 2022, at least seven U.S. states have proposed bills undermining the rights of transgender youth [30], including restricting access to school sports teams and school restrooms, and gender-affirming medical care (e.g., puberty blockers). The transgender stigma may even lead transgender or unsure youth in the future to conceal their gender identity [31].

Our study suggests that transgender and gender nonconforming (TGNC) students are both more vulnerable to bullying victimization than cisgender peers. TGNC students are also more likely to miss days of school [32] and experience poorer mental health outcomes [33]. The states and schools could implement policies that protect TGNC youth from these life adverse experiences. Anti-bullying and nondiscrimination policies on gender identity/expression are associated with lower prevalence of bullying victimization [34]. Besides, teacher training on gender identity/expression may be scaled up as it could help decrease school-based victimization [35].

### Limitations

The findings in this study are subject to at least four limitations. First, YRBS data are school-based, which means our findings might not be generalizable to out-of-school adolescents[6]. Given that transgender and gender non-conforming students are more likely to drop out than cisgender peers, this group may be underestimated herein[36]. Second, YRBS data are self-reported under conditions of assured confidentiality, so the experience of being bullied and gender identity/expression may be affected by the retrospective recall and social desirability biases. Third, YRBS data are cross-sectional, which hinders us to study the health of gender-fluid students whose gender identity varies over time and prevents us from discerning the directionality of the associations of GNC and gender minority with bullying victimization. Fourth, YRBS data did not include information on gender-based bullying victimization, general bullying indicators in YRBS might overestimate the risk of bullying victimization.

## Conclusions

Given the similarities and differences in bullying victimization between transgender identity and GNC, educators and parents should be more supportive of variety of gender identity and expression, especially for AMAB adolescents. Policymakers and child health advocates should make the programs more accessible and equitable for all the adolescents.

## Data Availability

Data is available at https://www.cdc.gov/healthyyouth/data/yrbs/index.htm.

## Abbreviations

GNC: Gender nonconformity
GC: Gender conformity
LGB: Lesbian, gay, bisexual
YRBS: Youth Risk Behavior Survey
APR: Adjusted prevalence ratio
CI: Confidence interval
ASAB: Assigned sex at birth
AFAB: Assigned female at birth
AMAB: Assigned male at birth
TGNC: Transgender and gender-nonconforming
